# Is there any association between adherence to the Mediterranean Diet and Dietary Total Antioxidant Capacity with Bacterial Vaginosis? Results from a Case–Control study

**DOI:** 10.1186/s12905-022-01833-8

**Published:** 2022-06-20

**Authors:** Morvarid Noormohammadi, Ghazaleh Eslamian, Seyyedeh Neda Kazemi, Bahram Rashidkhani

**Affiliations:** 1 Department of Nutrition, School of Public Health, Iran University of Medical Sciences, Tehran, Iran; 2 Student Research Committee, Faculty of Public Health Branch, Iran University of Medical Sciences, Tehran, Iran; 3 Department of Cellular and Molecular Nutrition, Faculty of Nutrition and Food Technology, National Nutrition and Food Technology Research Institute, Shahid Beheshti University of Medical Sciences, No 7, Hafezi St., Farahzadi Blvd., P.O. Box 19395-4741, Tehran, 1981619573 Iran; 4 Department of Obstetrics and Gynecology, School of Medicine, Preventative Gynecology Research Center, Imam Hossein Hospital, Shahid Beheshti University of Medical Sciences, Tehran, Iran; 5 Department of Community Nutrition, Faculty of Nutrition and Food Technology, National Nutrition and Food Technology Research Institute, Shahid Beheshti University of Medical Sciences, Tehran, Iran

**Keywords:** Mediterranean diet, Bacterial vaginosis, Inflammation, Oxidative stress, Case–control study

## Abstract

**Background:**

Bacterial vaginosis, BV, is a common inflammatory vaginal dysbiosis. The Mediterranean diet, MD, containing foods rich in antioxidant compounds, is shown to be beneficial for inflammatory conditions. This study aimed to investigate the association between MD adherence and dietary total antioxidant capacity (DTAC) with BV.

**Methods:**

This case–control study was conducted on 143 BV-affected and 151 healthy individuals aged between 15 and 45 years. The Amsel criteria were used to detect newly diagnosed patients with BV by a gynecologist. The 168-item food frequency questionnaire (FFQ) was used to record participants' dietary intakes in the last year. The reported data in the FFQ was used to measure adherence to the MD by calculating the Medi-Lite score and to measure the DTAC by calculating ferric-reducing antioxidant power, FRAP, based on the related databases. Logistic regression models were used to determine the association between Medi-Lite and DTAC and BV odds.

**Results:**

The highest tertile of Medi-Lite score was associated with a reduced odds of BV in the crude model (Odds Ratio, OR: 0.49, 95% Confidence Interval, 95% CI 0.25, 0.96, P for trend: 0.023). This significant inverse association was not observed in the last model adjusted for age, body mass index (kg/m^2^), waist circumferences (cm), cigarette smoking, frequency of pregnancy, and physical activity (MET/h/d). In crude and adjusted odels, BV odds decreased in the highest tertile of vegetable (adjusted OR, aOR: 0.32, 95% CI 0.16, 0.63, P for trend: 0.001), fish (aOR: 0.46, 95% CI 0.25, 0.84, P for trend: 0.009), legumes (aOR: 0.26, 95% CI 0.14, 0.50, P for trend < 0.001), and meat (aOR: 0.29, 95% CI 0.15, 0.56, P for trend < 0.001) groups. There was no significant association between DTAC and BV odds.

**Conclusions:**

The significant inverse association between the MD adherence and BV odds did not remain after modifying for confounders; besides, DTAC was not associated with BV odds. However, some of the MD components might be associated with a reduced odds of BV.

## Introduction

Nonspecific vaginitis (NSV), alternatively referred to as bacterial vaginosis, BV, is an inflammatory condition and the most prevalent form of vaginal dysbiosis [1], affecting between 5 and 70% of women worldwide [2]. A vaginal microbiota dominated by *Lactobacillus* spp. is healthier, and any diversity in vaginal microbiota results in dysbiosis; Such as a decrease in the number of *Lactobacillus* spp. and an increase in the number of opportunistic pathogens that are either not normally present in the human vaginal microbiota or are present in significantly lower numbers [3], like *Gardnerella vaginalis* [4]. As a result of this imbalance, many reactive oxidant species (ROS) are produced and accumulated [1]. So in comparison to healthy individuals, patients with BV may have a greater degree of oxidative stress in their vaginal discharge, as shown by a decrease in antioxidative enzymes, superoxide dismutase (SOD) and catalase (CAT), activity, and substantially increased hydrogen peroxide (H2O2) and malondialdehyde (MDA), a marker for lipid peroxidation in cells, levels [5]. The oxidative stress followed by increased intracellular levels of ROS causes damage to lipids, proteins, and DNA and leads to a variety of gynecological conditions [6], including fibroids, endometriosis, and postoperative adhesions [5]. Besides, a sufficient supply of antioxidants neutralizes excess oxidants during oxidative stress [7].

Mediterranean diet, MD, is a plant-based diet that emphasizes high-fiber foods such as fruit, vegetables, whole grains, legumes, moderate amounts of fish, white meat, alcohol, and less red meat and sweets. MD is characterized by a high monounsaturated fat-to-saturated fat ratio, with total fat accounting for 30–40% of daily calorie intake [8]. The MD has many foods high in antioxidant compounds, contributing to its advantages [9], such as a decrease in cardiovascular disease, breast cancer, colorectal cancer, diabetes, obesity, and inflammatory indicators [10]. There is a strong inverse link between the MD and plasma oxidative stress [11]. Among the major components of the MD, olive oil is shown to have antioxidant properties mainly due to being a good source of oleic acid [12] and polyphenols which reduce the ROS [13]. The MD contains additional sources of polyphenols, including vegetables, fruits, nuts, and legumes [14]. Nuts, broccoli, garlic, and grains are among the rich sources of selenium, an antioxidant trace element, in the MD [15]. The MD components also have synergistic effects; for example, carotenoids with vitamin E stimulate the antioxidant properties of vitamin C [16]. According to one research, greater adherence to the MD is linked with higher dietary total antioxidant capacity (DTAC) levels [17], an indicator that measures the overall antioxidant capacity of diet and demonstrates chronic inflammatory states [18].

Previous studies have reported the association between diet and BV odds [19–25]; however, due to the lack of data in this field, this study aimed to investigate the association between the MD and DTAC with BV odds.

## Materials and methods

### Ethical considerations

This study was approved by the National Nutrition and Food Technology Research Institute, Shahid Beheshti University of Medical Sciences, Tehran, Iran (the ethics committee code: IR.SBMU.NNFTRI.REC.1399.054). Before participating in the research, all individuals provided written informed permission.

### Study population

The present case–control study was conducted to examine the association between the MD and DTAC with odds of BV. The case–control studies' specific formula was used for the sample size calculation. Based on the reported data, 73% of Iranian adults had unhealthy dietary patterns [26]. The odds ratio (OR) for BV was assumed to be 2.5 among women with unhealthy dietary patterns relative to non-adherence. To attain statistical power equal to 80% and alpha error equal to 5%, a total of 125 BV-affected women (cases) and 125 healthy women (controls) were calculated and were determined to enter the study [27]. Participants were recruited via a convenience sample technique from patients referred to the Imam Hossein Hospital's gynecological clinic in Tehran, Iran, who expressed an interest in participating in the research. Inclusion criteria were considered age range of 15–45 years, absence of pregnancy, menopause, use of antibiotics, probiotics, hormonal contraceptives, vaginal douches, or immunosuppressive medications, and systemic illnesses, systemic Immunity diseases, chronic infection, diet-related chronic illnesses (diabetes, cardiovascular disease, etc.), or any condition of the uterine cavity, such as polyps and fibroids, and hysterectomy. The participants had no stature problems, as the height measures were taken, standing straight up. So, any participants with a problem in stature were excluded. Additionally, if at least 60% of the food frequency questionnaire items were not completed, the report of energy received was excluded from the ± 3 standard deviation (SD) from the average energy intake, and the desire to leave the study for any reason, individuals were excluded from the study.

### Medical assessments

A gynecologist checked all individuals to rule out BV. For the clinical diagnosis of BV, the Amsel criteria were used, which require the presence of at least three of four of the following: homogeneous and a dilute vaginal discharge, vaginal pH greater than 4.5, presence of 20% of clue cells under saline microscopy, and fish odor after adding 10% potassium hydroxide to the discharge slide [21, 28–30].

### Data and sample collection

At baseline, participants completed comprehensive questionnaires on demographic information and food frequency. This food frequency questionnaire (FFQ) was valid and reliable [31], containing 168 food items that each have a standard serving size according to the Willet method [32] and was developed and has been utilized in earlier research [33, 34] to determine dietary patterns. The average size of each food item in the FFQ was explained to the case and control groups during the interview, and then they were questioned about their frequency of intake of each food item during the previous year.

After completing the FFQ, the food values were converted to grams using the home scale instructions. Following that, the subjects' weight, height, and waist circumference (WC) were determined, weight in light clothes with a precision of 100 g and height in a standing and straight posture with a ruler put on the individual's head, without shoes, and with the shoulders in a normal position, with a precision of one millimeter. The body mass index (BMI) was calculated by dividing the weight (kg) by the height (square meters) square. All participants under research answered questionnaires and had their anthropometric indices assessed by a questioner who had undergone the required training before beginning the study. The person conducting the diagnostic BV test was unaware of the patients' exposure circumstances (food consumption) to minimize selection bias. Additionally, to minimize information bias, case group samples were drawn from patients newly diagnosed with BV, and questionnaires were completed by a trained interviewer who was unaware of the result of the samples at the time of the interview. Additionally, diagnostic tests were done at a hospital laboratory by a person.

### Medi-Lite

MD adherence was calculated using a validated procedure called Medi-Lite [35]. For food items typical of the MD (fruits and nuts, fish, vegetables, legumes, whole grains, and ratio of monounsaturated fatty acid (MUFA) to saturated fatty acid (SFA)), a value of 2 was assigned to the highest tertile of consumption, 1 to the middle tertile, and 0 to the lowest tertile. In comparison, food categories that are not characteristic of the MD (meat and dairy products) were given a value of 2 for the lowest tertile of consumption, 1 for the middle tertile, and 0 for the greatest tertile of consumption. Due to a data shortage in our dataset, alcohol consumption was not included in the current study's score calculation. The total score was calculated by adding these values together. It ranges from 0 (poor adherence) to 16 (great adherence) (high adherence) [35]. All dietary groups had been adjusted for total energy intake using the residual method.

### DTAC

DTAC was measured using published databases that provided the antioxidant capacity in foods by assays ferric-reducing antioxidant power (FRAP). Food items were adjusted for total energy intake using the residual method [36].

### Statistical analysis

SPSS software version 22 was used for statistical analysis. Statistical significance was defined as a P-value less than 0.05 for all hypothesis tests. Kolmogorov–Smirnov tests and Q–Q plots were used to ensure that continuous variables had a normal distribution. The median (interquartile range IQR, frequency, and percentages were used to indicate general quantitative and categorical features, respectively. To compare qualitative characteristics between case and control groups, the Chi-Square test was employed. Additionally, the Student t-test was employed to evaluate quantitative confounding factors across case and control groups, whereas the Mann–Whitney test was utilized when quantitative variables were not normally distributed. To evaluate quantitative confounding factors across tertiles of Medi-Lite, the one-way ANOVA test was used. Logistic regression was used to estimate the crude and adjusted odds ratios (aOR) with 95% confidence intervals (CIs) to analyze the link between Medi-Lite and DTAC and BV odds. aORs were obtained by controlling for age, BMI (kg/m^2^), WC (cm), cigarette smoking, frequency of pregnancy, and physical activity (MET/h/d).

## Results

Table 1 summarizes the general characteristics of the cases (n = 143) and controls (n = 151). The median (Q1-Q3) age of individuals in cases and controls was 30 (25–33) and 32 (24–37) years, respectively. In both the case and control groups, there was a significant difference in the age of various Medi-Lite score tertiles. Additionally, there was a significant difference in the number of cigarettes smoked per day and the frequency of pregnancy in the tertiles of the case group's Medi-Lite score. Cases had a substantially higher median (Q1–Q3) BMI (26.1 (23.4–28.7)) than the control group (24.6 (22.2–28.0)). Additionally, BV patients had a substantially greater median (Q1–Q3) WC than controls (85 (76–95) vs. 82 (74–90)). There were no statistically significant differences between cases and controls regarding other demographic and lifestyle factors.

**Table 1:** General characteristics of participants with bacterial vaginsis and healthy controls separately across tertiles of Medi-Lite

Characteristic^s^	Control				Case			
	Tertile 1 n = 72	Tertile 2 n = 47	Tertile 3 n = 32	P value^b^	Tertile 1 n = 89	Tertile 2 n = 36	Tertile 3 n = 18	P value^b^
Age, year, median (Q_1_–Q_3_)	28 (24–35)	33 (25–39)	33.5 (30–37.8)	0.023*	28 (24–33)	31 (29–33.8)	32 (30–37)	0.004*
Familial history of BV, yes	15 (20.8)	13 (27.7)	9 (28.1)	0.605	50 (56.2)	18 (50)	9 (50)	0.772
Education				0.939				0.367
Primary/secondary school	18 (25)	11 (23.4)	10 (31.3)		20 (22.5)	11 (30.6)	5 (27.8)	
Undergraduate	34 (47.2)	23 (48.9)	13 (40.6)		45 (50.6)	21 (58.3)	10 (55.6)	
Graduate	20 (27.8)	13 (27.7)	9 (28.1)		24 (27)	4 (11.1)	3 (16.7)	
Cigarette per day				0.475				0.002*
0	71 (98.6)	47 (100)	31 (96.9)		65 (73)	36 (100)	17 (94.4)	
1–2	0	0	0		12 (13.5)	0	0	
≥ 3	1 (1.4)	0	1 (3.1)		12 (13.5)	0	1 (5.6)	
Employment status, employed	18 (25)	12 (25.5)	12 (37.5)	0.387	26 (29.2)	13 (36.1)	4 (22.2)	0.553
Monthly income, < 250US$	59 (81.9)	38 (80.9)	24 (75)	0.707	71 (79.8)	27 (75)	12 (66.7)	0.418
Frequency of pregnancy				0.681				0.037*
0	37 (51.4)	21 (44.7)	12 (37.5)		39 (43.8)	18 (50)	6 (33.3)	
1–2	27 (37.5)	21 (44.7)	17 (53.1)		44 (49.4)	16 (44.4)	6 (33.3)	
≥ 3	8 (11.1)	5 (10.6)	3 (9.4)		6 (6.7)	2 (5.6)	6 (33.3)	
Menstrual cycle, regular	46 (63.9)	35 (74.5)	21 (65.6)	0.468	59 (66.3)	26 (72.2)	9 (50)	0.264
Number of sexual partners in the previous month				0.427				0.300
0	29 (40.3)	16 (34)	8 (25)		34 (38.2)	9 (25)	5 (27.8)	
1	41 (56.9)	29 (61.7)	24 (75)		53 (59.6)	24 (66.7)	12 (66.7)	
≥ 2	2 (2.8)	2 (4.3)	0		2 (2.2)	3 (8.3)	1 (5.6)	
Physical activity (MET/h/d), median (Q_1_–Q_3_)	40.5 (36.4–45.7)	40.1 (35.8–46.9)	39.2 (36.2–46.4)	0.890	39.8 (35.2–45)	39.9 (36.8–44.6)	40.8 (34.7–43.8)	0.786
BMI, (kg/m^2^), median (Q1–Q3)	24.3 (22.2–26.7)	24.6 (22–29.1)	24.3 (22.1–30.9)	0.120	26 (22.5–29.1)	27.2 (24.5–28.4)	26.6 (23.4–28.3)	0.724
Waist circumference (cm), median (Q_1_–Q_3_)	80 (72.5–90)	83 (75–100)	84 (71.3–94)	0.323	87 (80–95.5)	84.5 (64–91.5)	85.5 (46–100.8)	0.032*
Supplements								
Calcium 500 mg/day, yes	13 (18.1)	8 (17)	3 (9.4)	0.518	5 (5.6)	2 (5.6)	3 (16.7)	0.223
Folate 400 μg/day, yes	12 (16.7)	5 (10.6)	5 (15.6)	0.660	18 (20.2)	8 (22.2)	4 (22.2)	0.956
Vitamin D 50,000 IU/month, yes	29 (40.3)	20 (42.6)	17 (53.1)	0.467	31 (34.8)	15 (41.7)	3 (16.7)	0.186
Iron 30 mg/day, yes	21 (29.2)	15 (31.9)	5 (15.6)	0.242	16 (18)	7 (19.4)	3 (16.7)	0.950
Omega-3 1–3 g/day, yes	3 (4.2)	3 (6.4)	2 (6.3)	0.839	2 (2.2)	4 (11.1)	1 (5.6)	0.078

BV, Bacterial Vaginosis; BMI, Body Mass Index

^a^Values are No (%)unless otherwise noted

^b^Using Mann–Whitney U or χ^2^ test/Fisher's exact test, as appropriate

The association between the Medi-Lite score and BV odds is shown in Table 2. The highest tertile of the Medi-Lite score (equals more than 10) and the fruits and nuts group intake (equals more than 500 gr/day from the sum of fruits and nuts) were negatively related to BV in a crude model (P for trend: 0.023, and 0.013, respectively). In the adjusted model, the odds of BV in the highest tertile of Medi-Lite score were 47% lower than in the reference, but this association was of borderline statistical significance (aOR: 0.53, 95% CI 0.26, 1.08, P for trend: 0.085). There was a significant diverse association between the highest tertile of vegetables (equals to more than 367 gr/day, aOR: 0.32, 95% CI 0.16, 0.63, P for trend: 0.001), fish (equals to more than 10 gr/day, aOR: 0.46, 95% CI 0.25, 0.84, P for trend: 0.009), and legumes (equals to more than 48 gr/day, aOR: 0.26, 95% CI 0.14, 0.50, P for trend < 0.001) and BV odds. However, participants in the last tertile of meat consumption (equal to more than 100 gr/day) had lower odds of BV (aOR: 0.29, 95% CI 0.15, 0.56, P for trend < 0.001) in both crude and adjusted models. As shown in Table 3, there was no significant association between DTAC and BV odds.

**Table 2:** Adjusted odds ratio (OR) estimates and 95% confidence intervals (CIs) for bacterial vaginosis according to the tertile of Medi-Lite*

	Tertiles of Medi-Lite			*P* for trend
	1st	2nd	3rd	
Medi-Lite score (energy-adjusted)				
No. cases/no. controls	89/72	36/47	18/32	
Base model^†^	1.00 (Ref.)	0.65 (0.38–1.12)	0.49 (0.25–0.96)	0.023
Full model^‡^	1.00 (Ref.)	0.81 (0.46–1.43)	0.53 (0.26–1.08)	0.085
Fruits and nuts (energy-adjusted, g/day)				
No. cases/no. controls	66/50	46/51	31/50	
Base model^†^	1.00 (Ref.)	0.71 (0.41–1.22)	0.48 (0.27–0.86)	0.013
Full model^‡^	1.00 (Ref.)	0.90 (0.51–1.61)	0.60 (0.33–1.11)	0.112
Vegetable (energy-adjusted, g/day)				
No. cases/no. controls	73/50	50/51	20/50	
Base model^†^	1.00 (Ref.)	0.69 (0.40–1.17)	0.29 (0.15–0.55)	< 0.001
Full model^‡^	1.00 (Ref.)	0.71 (0.40–1.25)	0.32 (0.16–0.63)	0.001
Fish (energy-adjusted, g/day)				
No. cases/no. controls	74/50	38/51	31/50	
Base model^†^	1.00 (Ref.)	0.49 (0.28–0.86)	0.41 (0.23–0.74)	0.002
Full model^‡^	1.00 (Ref.)	0.54 (0.30–0.97)	0.46 (0.25–0.84)	0.009
Legumes (energy-adjusted, g/day)				
No. cases/no. controls	82/50	42/51	19/50	
Base model^†^	1.00 (Ref.)	0.53 (0.31–0.91)	0.23 (0.12–0.44)	< 0.001
Full model^‡^	1.00 (Ref.)	0.59 (0.33–1.05)	0.26 (0.14–0.50)	< 0.001
Whole grains (energy-adjusted, g/day)				
No. cases/no. controls	54/50	59/51	30/50	
Base model^†^	1.00 (Ref.)	1.07 (0.63–1.84)	0.59 (0.32–1.08)	0.111
Full model^‡^	1.00 (Ref.)	1.11 (0.63–1.96)	0.54 (0.28–1.02)	0.082
The ratio of MUFA to SFA (energy-adjusted)				
No. cases/no. controls	39/50	64/51	40/50	
Base model^†^	1.00 (Ref.)	1.65 (0.94–2.89)	1.04 (0.58–1.89)	0.895
Full model^‡^	1.00 (Ref.)	1.55 (0.86–2.81)	1.01 (0.53–1.92)	0.994
Meats (energy-adjusted, g/day)				
No. cases/no. controls	77/50	45/51	21/50	
Base model^†^	1.00 (Ref.)	0.58 (0.34–1.00)	0.27 (0.14–0.51)	< 0.001
Full model^‡^	1.00 (Ref.)	0.60 (0.34–1.05)	0.29 (0.15–0.56)	< 0.001
Dairy products (energy-adjusted, mg/day)				
No. cases/no. controls	50/50	51/51	42/50	
Base model^†^	1.00 (Ref.)	0.99 (0.57–1.73)	0.81 (0.45–1.43)	0.467
Full model^‡^	1.00 (Ref.)	0.89 (0.49–1.61)	0.62 (0.33–1.17)	0.144

*Logistic regression model

^†^Adjusted for age

^‡^Adjusted for age, BMI (kg/m^2^), WC (cm), Cigarette per day, Frequency of pregnancy and physical activity (MET/h/d)

**Table 3:** Adjusted odds ratio (OR) estimates and 95% confidence intervals (CIs) for bacterial vaginosis according to the tertile of FRAP*

	Tertiles of FRAP			*P* for trend
	1st	2nd	3rd	
FRAP score (energy-adjusted)				
No. cases/no. controls	46/50	42/51	55/50	
Base model^†^	1.00 (Ref.)	0.88 (0.50–1.57)	1.18 (0.68–2.06)	0.538
Full model^‡^	1.00 (Ref.)	0.70 (0.38–1.28)	0.99 (0.55–1.78)	0.983

*Logistic regression model

^†^Adjusted for age

^‡^Adjusted for age, BMI (kg/m^2^), WC (cm), Cigarette per day, Frequency of pregnancy and physical activity (MET/h/d)

## Discussion

This study showed an inverse association between MD adherence and odds of BV in the crude model. However, BV odds was not associated with the DTAC. Based on our knowledge, this was the first investigation of the association between the MD and DTAC with BV odds.

In this study, a significant negative association between the vegetables group and odds of BV in both crude and adjusted models was observed. Also, in the crude model, the odds of BV were decreased in the highest tertile of fruits and nuts group.

According to Parsapure et al., educating patients about the importance of increasing their vegetable and fruit consumption may play a significant role in treating vaginitis [37].

Vegetables and fruits are natural sources of non-digestible oligosaccharides, prebiotics, and functional carbohydrates that benefit intestinal health [38]. Functional foods, probiotics, and prebiotics have been shown to be helpful in the treatment of vaginal inflammation [25, 39].

Neggers et al. investigated a significant negative correlation between severe BV and folate and vitamin E intake [24]. The primary sources of folate are fruits and vegetables, particularly green vegetables [40]. Vegetable oils, fresh fruits, vegetables, and nuts are also sources of vitamin E intake [41]. Vitamin E is a potent antioxidant affecting the immune system [42–44]. Both folate and vitamin E consumption may enhance immunological function, leading to a lower risk of severe BV [24].

The tomato is a typical MD vegetable that contains antioxidants. Lycopene, a carotene phytochemical found in tomatoes, stimulates the activity of antioxidant enzymes (SOD, GPX, and catalase) and has anti-inflammatory characteristics [45]. On the other hand, women are more susceptible to BV infection if they are deficient in micronutrients such as vitamins A, E, D, C, and beta-carotene, a sign of low fruit and vegetable consumption. In recent years, scientists discovered that a nutrient-dense diet reduces the chance of apparent bacterial infection. Regular intake of fresh fruit and vegetables decreases the risk of vaginitis [24, 37, 46]. Betaine is another chemical found in some vegetables, such as spinach. According to Tuddenham et al., reduced betaine consumption is linked with an increased risk of molecular-BV [21].

Additionally, Shivakoti et al. found a link between energy-adjusted total fiber intake and a decreased risk of BV [22]. Vegetables and fruits are a great source of fiber [47], and numerous prebiotic fibers promote monocultures of the main *Lactobacillus* species that dominate the vaginal microbiota while not affecting BV-related bacteria *G. vaginalis* [48]. Besides, the rectum may act as a source of vaginal colonization [49], and dietary fiber may influence the intestinal barrier integrity, microbial translocation, and inflammation [50–53]. In addition, obese individuals consume fewer fruits and vegetables [54], and according to Brookheart et al., BV is associated with obesity [23]. In Thoma et al.'s study, glycemic load (GL) was shown to enhance the persistence and acquisition of BV because of the effect of the high-GL diets on the host's response to bacterial colonization and increased oxidative stress and decreased immunological function [25]. However, as shown by Lau et al., beneficial components of fruits and vegetables (e.g., fiber) may attenuate the adverse effects of high glycemic load foods like insulin resistance [55].

Flavonoids are anti-inflammatory chemical compounds naturally found in citrus fruits and vegetables that inhibit transcription factors such as Nuclear factor-κB (NF-κB) [56]. NF-κB is the distinguishing factor involved in the pro-inflammatory signaling pathways of numerous Toll-like receptors, a large family of pattern recognition receptors (PRRs) that respond specifically to various pathogens. NF-κB stimulation in various cell types has been observed in vaginal secretions of BV-infected women [3].

In this study, in both crude and adjusted models, the odds of BV decreased in the highest tertile of meat, legumes, and fish groups. Verstraelen et al. have shown a strong and independent association between subclinical iron insufficiency and vaginosis-like microbiota during early pregnancy, presumably due to insufficient pre-conceptional iron supply [57]. Iron deficiency may alter innate and adaptive immune system responses of the vaginal mucosa leading to infection disease susceptibility [58, 59]. Animal meat is the richest and most bioavailable source of dietary heme iron [60]. Heme iron is estimated to make up 50%, 40%, and 20% of the iron in meat, poultry, and fish, respectively [61]. Legumes are also a great source of iron [62]. Inversely, intake of processed meats and fast foods is associated with higher BV odds, as has been observed in another study [20], maybe because of the high load of fat in these foods and the positive association between total fat consumption and increased risk of BV and severe BV [24].

## Strengths and limitations

The present research has some strengths in many ways. Participants who reported their energy consumption incorrectly or excessively were removed. Patients with newly diagnosed BV were included. Selecting incident cases would bolster a causal interpretation and mitigate recall bias [63, 64]. Both cases and controls had high participation rates. Numerous possible variables were available for adjustment in regression models. A single individual conducted a diagnostic test in the hospital laboratory to eliminate information bias in all groups. Additionally, the questions were completed by a professional dietitian who was blinded to the diagnostic findings during the interview.

The present research, however, has considerable drawbacks. While researchers made every effort to avoid bias, selection bias, measurement bias, and recall bias may result in misleading results when using a case–control design. The present research did not examine the different bacteria that cause BV. The Nugent score, the gold standard for BV diagnosis [29], was not used. Alcohol and opium data were not gathered due to the Iranian cultural and religious prohibition.

To the best of our knowledge, the current study was the first which investigated the association between MD, DTAC, and BV; however, further clinical trials and prospective studies are needed to confirm the results.

## Conclusion

Taken together, these results indicate that following the Mediterranean dietary pattern, high in vegetables, fish, and legumes, may be associated with reduced BV odds and is suggested for the woman at high risk of BV. However, DTAC seems to be unrelated to BV odds.

## Data Availability

The data that support the findings of this study are available from the corresponding author, but restrictions apply to the availability of these data, which were used under license for the current study, and so are not publicly available. However, data are available from the authors upon reasonable request and with the corresponding author's permission.

## Abbreviations

NSV: Nonspecific vaginitis
BV: Bacterial Vaginosis
ROS: Reactive oxidant species
SOD: Superoxide dismutase
CAT: Catalase
MDA: Malondialdehyde
MD: Mediterranean diet
DTAC: Dietary total antioxidant capacity
FFQ: Food frequency questionnaire
WC: Waist circumference
BMI: Body mass index
MUFA: Monounsaturated fatty acid
SFA: Saturated fatty acid
FRAP: Ferric-reducing antioxidant power
